# Roles of increased NUCKS1 expression in endometriosis

**DOI:** 10.1186/s12905-023-02563-1

**Published:** 2023-08-15

**Authors:** Bo Li, Bocen Chen, Xiaoli Wang, Man Xiao, Kan Zhang, Wenjiao Ye, Da Zhao, Xiaohua Wang, Yan Yu, Jun Li, Xun Xu, Wenhui Zhang, Yanhua Zhang

**Affiliations:** 1 Hainan Women and Children’s Medical Center, Hainan, China; 2 Key Laboratory of Biochemistry and Molecular Biology, Hainan Medical University, Hainan, China; 3 Gansu University of Traditional Chinese Medicine, Lanzhou, China; 4 Gansu Provincial Maternal and Child Health Hospital, Lanzhou, China

**Keywords:** NUCKS1, Endometriosis, Phosphatidylinositol 3-Kinase, NF-kappa B

## Abstract

**Background:**

Endometriosis is still a difficult problem for women. The Nuclear Ubiquitous Casein and cyclin-dependent Kinase Substrate 1 (NUCKS1) gene is located on human chromosome 1q32.1. It encodes the NUCKS1 protein, a 27 kDa nuclear DNA binding protein that plays an important role in cell growth and proliferation. NUCKS1 plays an important role in the development of many diseases. However, its role in endometriosis is unclear.

**Methods:**

Ectopic endometrial tissues and normal tissue specimens were collected, and the expression of NUCKS1, NF-κB and PI3K was detected by RT-qPCR and immunohistochemistry. Inhibition of NUCKS1 in hEM15A cells, study the changes in cell viability, apoptosis, migration and protein expression by CCK8 assay, flow cytometry, wound-healing assay, western blot and ELISA techniques. The comparison of differences between the two groups was implemented using unpaired sample t test or Mann-whitney U test. One-way analysis of variance or Kruskal-wallis test was used for comparisons among the three groups.

**Results:**

(1) NUCKS1 is highly expressed in endometriosis tissues. (2) Inhibition of NUCKS1 decreases cell viability and capability of migration, and increases apoptosis in endometriosis cells. (3) Expressions of NF-κB and PI3K are increased in endometriosis tissues, and inhibition of NUCKS1 decreases the expression levels of PI3K and NF-κB in endometriosis cells. (4) Inhibition of NUCKS1 decreases the expression of VEGF.

**Conclusion:**

(1) NUCKS1 is overexpressed in endometriosis, and inhibition of NUCKS1 inhibits cell viability and capability of migration, and increases apoptosis. (2) NUCKS1 promotes the progress of endometriosis through activating PI3K and NF-κB pathways, and VEFG is also involved in this process.

**Supplementary Information:**

The online version contains supplementary material available at 10.1186/s12905-023-02563-1.

## Introduction

Endometriosis is still a difficult problem for women. It has the characteristics of implantation, infiltration and high recurrence rate, because it has several biological behaviors similar to malignant lesions, including cell invasion, neo-angiogenesis, and decreased apoptosis [1, 2]. Endometriosis (EMs) is a common gynecologic disease with an overall prevalence of about 10–15%, of which most patients are detected in the reproductive age and the mechanisms of which have not been fully elucidated [3]. Endometriosis not only causes pain, but also has serious consequences such as infertility [4]. How to effectively prevent and treat EMs and protect the fertility of EMs patients in their reproductive years is a great challenge for current clinical work. Therefore, it is particularly important to study the pathogenesis of EMs, prevent and treat EMs, and enhance the fertility of women in their reproductive age.

The Nuclear Ubiquitous Casein and cyclin-dependent Kinase Substrate 1 (NUCKS1) gene is located on human chromosome 1q32.1 [5] and encodes the NUCKS1 protein, a 27 kDa nuclear DNA binding protein that plays an important role in cell growth and proliferation [6]. NUCKS1 plays an important role in non-neoplastic diseases such as Parkinson disease [7] and bipolar disorder [8]. Moreover, NUCKS1 is highly expressed in a variety of malignancies and acts as a tumor-associated protein regulating cellular transcription and repair [9, 10]. However, its role in endometriosis is unclear. While endometriosis has similar biological characteristics to tumor, specifically, it has the characteristics of invasion, migration and susceptibility to recurrence. Therefore, it is worthwhile to explore whether NUCKS1 expression is as different in endometriosis as in tumors.

In this study, We mainly verified that NUCKS1 expression was increased in Endometriosis. Inhibition of NUCKS1 resulted in decreased cell viability and migration capacity and increased apoptosis. Meanwhile, NUCKS1 act through PI3K and NF-κB signaling pathways and cause increased expression of VEGF in endometriosis.

## Materials and methods

### Patient samples

Thirteen cases of ectopic endometrial tissues and two cases of eutopic endometrial tissues from patients treated at the Women and Children’s Center of Hainan Province from October 2020 to October 2021 in surgical patients with pathologically confirmed ovarian endometriosis were collected. Four cases of normal endometrial tissues were also collected for outpatient hysteroscopy. The inclusion and exclusion criteria of participants are as followed:

Inclusion criteria:

① General case data were perfect.

② Initial diagnosis of the experimental group was considered ovarian endometriosis and all were treated for the first time;

③ Control group had no patients with benign endometrial lesions or malignant endometrial lesions.

④ Patients were treated surgically after admission and had clear postoperative paraffin pathology diagnosis.

Exclusion criteria:

① The experimental group had patients with both ovarian endometriosis and other ovarian cysts.

② Patients in the control group had abnormal uterine bleeding or ovarian tumors producing estrogen, or a long history of estrogen or progesterone, or had malignant tumors.

③ Postoperative pathological diagnosis was missing.

All surgical specimens were taken immediately after isolation and part of the tissues were frozen in liquid nitrogen for experimental use, and the remaining tissues were sent to pathology.The study was approved by the hospital ethics committee, written informed consent was obtained from the patients for the use of all specimens, patient privacy was strictly protected, the use of tissue specimens outside this study was prohibited, and specimens were collected in accordance with the World Medical Association Declaration of Helsinki.

### Real-time fluorescence quantitative PCR (RT-qPCR)

Primer design Specific primers were designed according to the NUCKS1 sequence in the gene library using the software Primer Primier 5.0 (Premier Biosoft International, Palo Alto,CA, USA). The caretaker gene used GAPDH. Forward and reverse primers were: 5’-GGCCTGTCAGAAATAGGAAGGT-3’ and 5’-TTTAGCTTCTCGGGGAG ATGAT-3’ for human NUCKS1 promoter.

For tissue or cellular total RNA extraction, the Eastep®Super Total RNA Extraction Kit (LS1040) was used. RNA was reverse transcribed to cDNA using Hifair® II Reverse Transcriptase (YEASEN 11110ES92) according to the instructions. Hieff UNICON® qPCR SYBR Green Master Mix (Antibody method, Low Rox) (YEASEN 11199ES08) was used to detect gene expression using a Quantagene q225 fluorescence PCR instrument (NovoHozyme).

RT-qPCR data were used to calculate the relative expression using the 2^−ΔΔCt^ method.

### Immunohistochemistry (IHC)

Tissue was prepared by paraffin, dewaxed and hydrated by gradient of xylene and alcohol, and antigen repair solution was added to expose antigen sites. After blocking 10% goat serum at 37℃ for 30 min, CXCL9 antibody was added in a wet box overnight at 4℃, the excess antibody was washed off with PBS, and the secondary antibody was incubated with DAB for 30 min at room temperature. The slides were dehydrated with alcohol, transparent with xylene, sealed with neutral resin, and observed in bright field under an inverted microscope. Primary anti-NF-κB, anti-NUCKS1, Anti-PI3K and secondary antibodies were obtained from Thermo Fisher Scientific.

### Cell culture

Immortalized cells of eutopic endometrial mesenchymal cells from human endometriosis patients (hEM15A cell) as purchased from CCTCC (Wuhan,China). They were cultured in DMEM:F12 (Gibco A4192001) and 10% FBS (Gibco A3161001C) medium, 5% CO_2_ and 37 °C in a cell culture incubator. The cells were ready for passaging when they reached 70% fusion.

### Cell transfection

SiRNA provided by Integrated Biotech Solutions company (Shanghai, China). The hEM15A cells were seeded in six-well plates and grown in a 37℃ incubator overnight for transfection when the cell density reached 40%. Transfection reagents Lipo6000TM (Beyotime) was used for cell transfection.

### CCK-8 assay

The adenovirus-transfected successful hEM15A cells were digested and centrifuged for resuspension, and after calculating the concentration cells were inoculated according to a 96-well plate with 1500 cells per well and cultured overnight in a cell culture incubator at 5% CO2 and 37 °C. Then each well was spiked with CCK-8 reagent (Dojindo CK04) 10µL/well. The incubation was continued in the incubator at 37℃, and the OD values of each time period were measured at 450 nm after 1 h, 2 h, 3 and 4 h with an enzyme marker.

### Flow cytometry

hEM15A cells successfully transfected with adenovirus were digested with EDTA-free trypsin (Gibco 15,050,065). After digestion until the cells started to loosen and could be shed by shaking, the trypsin was aspirated and complete medium was added to terminate the digestion, and the cells were blown down with cell supernatant to try to disperse the cells into individual cells and transferred into EP tubes. Apoptosis detection was performed using Annexin V-FITC Apoptosis Detection Kit (YEASEN 40302ES60) and flow cytometry technology.

### Wound-healing assay

Cells were inoculated uniformly in 6-well plates and incubated overnight. When the cells are fused to about 80% of the monolayer, use the tip of a 200 µl pipette to make a “one-shape” mark at the bottom of the well plate and slide from one end to the other. Remove the old medium, wash gently with an appropriate amount of PBS to remove the suspended cells, and replace the medium with normal medium to continue the culture. After scratching, we took pictures in microscope and observed the healing of the scratch every 12 h. We selected the time point when the migration ability of each group was significantly different to take pictures.

### Western blot analysis

The cell samples were collected and lysed by adding RIPA lysis solution (YEASEN 20101ES60 ), protease inhibitor PMSF (YEASEN 20104ES03) (containing protease inhibitor) in proportion. Centrifuge for 10 min and collect the supernatant for protein quantification (BCA method) (Beyotime P0010S). The collected protein samples were sampled, electrophoresed, transferred to the membrane, rinsed, closed, incubated with primary and secondary antibodies, and developed by ECL luminescence kit (Beyotime P0018S). Our primary antibodies are listed below: PI3K rabbit pAb (#ab302958, 1:500, abcam, USA), AKT rabbit pAb (#4691, 1:1000, CST, USA), GAPDH rabbit pAb (#2118S, 1:1000, CST, USA). After incubation at 4 °C overnight, membranes were washed and incubated with appropriate secondary antibody (1:1000, Biyuntian, China). The blotted proteins were detected using ECL ( Thermo Fisher, USA ) reagents.It is worth noting that due to the different size of protein bands of the target gene, we cut them before the heteroclonal antibody, and have retained the clearest picture for this purpose.

### Enzyme-linked immunosorbent assay (ELISA)

The cell suspension of each group was diluted with PBS, and the cell concentration reached 1 million /ml. Repeated freezing and thawing with liquid nitrogen to destroy cells and release their contents. The supernatant was collected by centrifugation and then detected by human vascular endothelial cell growth factor (VEGF-A) ELISA kit (Shanghai Sangon Bioengineering D711056).

### Statistical analysis

Statistical analysis was implemented by using SPSS 18.0 and GraphPad 6.0. The data of the studied participants were checked for normal distribution using the Shapiro-Wilk method and the normal distribution tables or curves were submitted as Supplementary files within the submitted revised version of the manuscript. The differences between the two groups were analyzed by the unpaired sample t test (normal distribution) and Mann-whitney U test (non-normal distribution). The differences among the three groups and above were compared by One-way analysis of variance (normal distribution) and Kraskal Wall’s test (non-normal distribution). And differences were considered statistically significant when the *p* value was less than 0.05. ImageJ was used to quantitatively analyze the optical density and gray level of IHC images.

## Results

### NUCKS1 is overexpressed in endometriosis

**Fig. 1:**
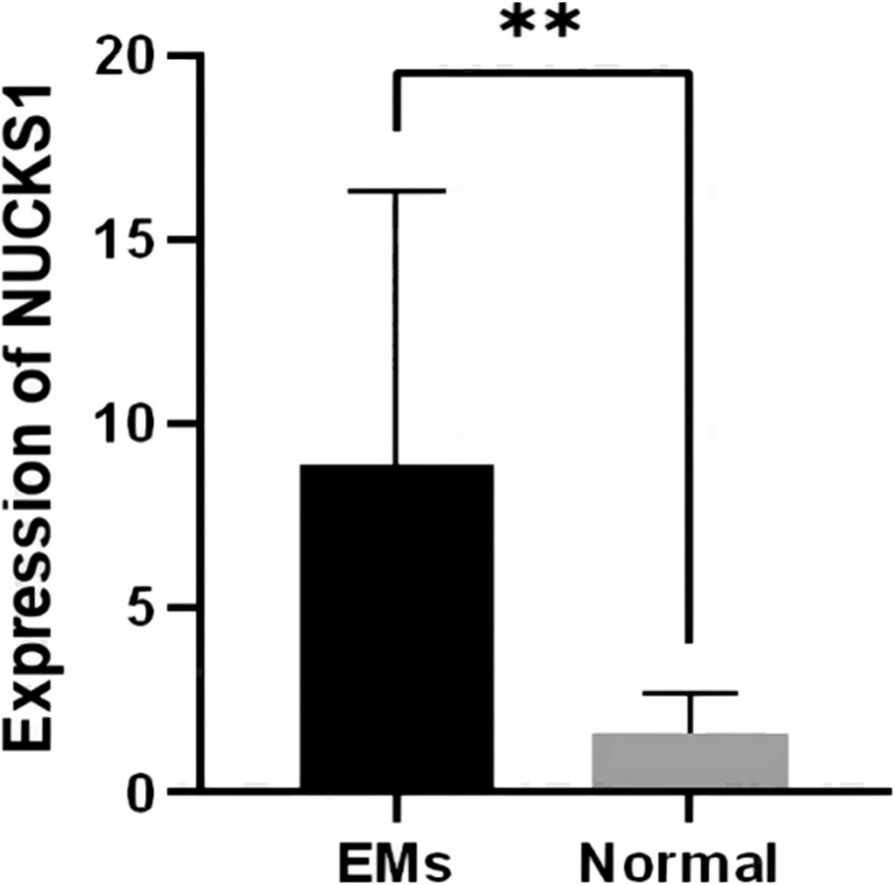
Expression of NUCKS1 mRNA levels in endometriosis tissue and normal tissue. （EMs n=11，Normal n=6. Not consistent with normal distribution. Differences between the two groups were tested by Mann-whitney U test. **P < 0.01)

**Fig. 2:**
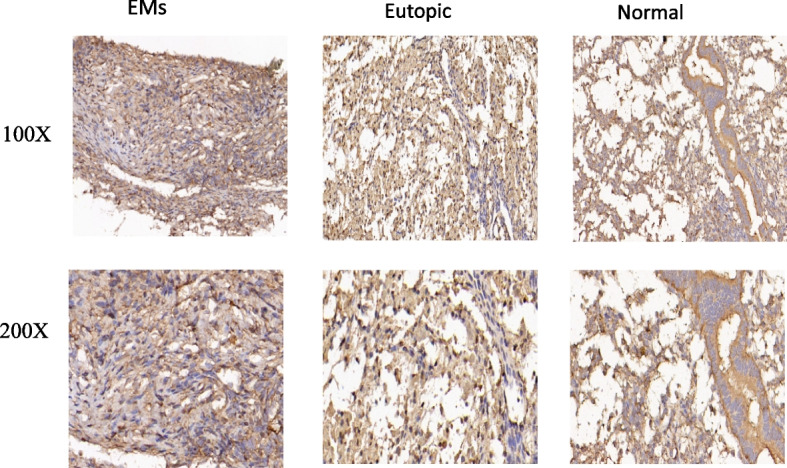
Immunohistochemical staining of NUCKS1 in endometriosis tissue, eutopic tissue and normal tissue

By RT-qPCR analysis of 11 patients and 6 control samples (2 cases of ectopic endometrial tissue and 4 cases of normal endometrium tissue samples), We found NUCKS1 expression in ectopic, eutopic and normal endometrium tissues, but NUCKS1 expression in ectopic endometrial tissues was significantly higher than the eutopic and normal endometrium (Fig. 1). This result was also demonstrated by assessing NUCKS1 expression by immunohistochemistry of tissues and immunohistochemistry also showed that NUCKS1 was mainly located in cytoplasm (Figure 2).

### After inhibition of NUCKS1, hEM15A cells have decreased cell viability, increased apoptosis, and decreased capacity of migration

**Fig. 3:**
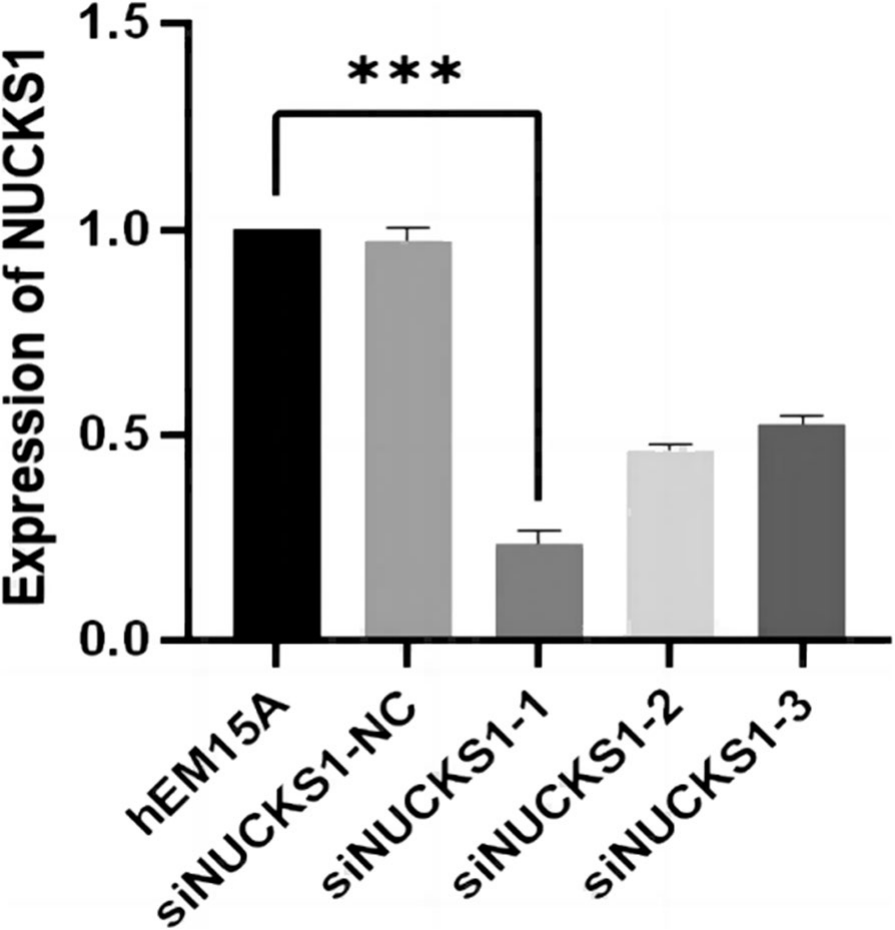
The expression of NUCKS1 mRNA levels in cells transfected with different siNUCKS1.(*n*=3. Not consistent with normal distribution. Differences between groups were compared using Kraskal Wall's test. ***P  < 0.001)

**Fig. 4:**
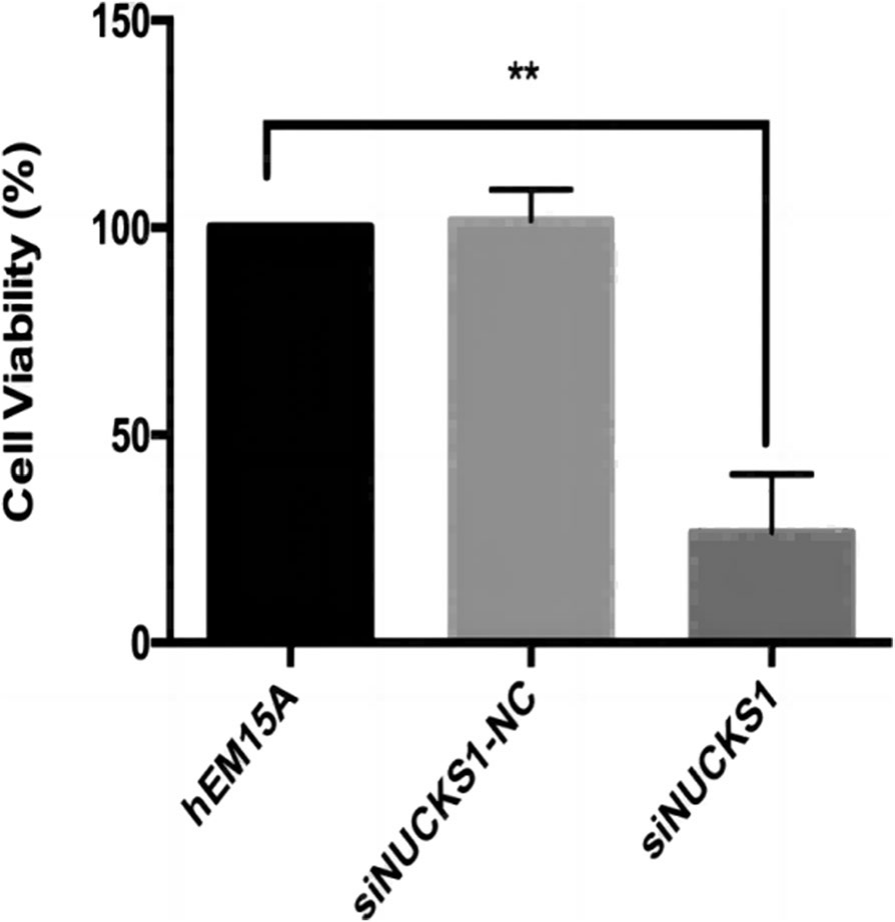
The cell viability of each group cells after transfection of siNUCKS1.(n=3. Not consistent with normal distribution. Differences between groups were compared using Kraskal Wall's test. ** *P* < 0.01)

**Fig. 5:**
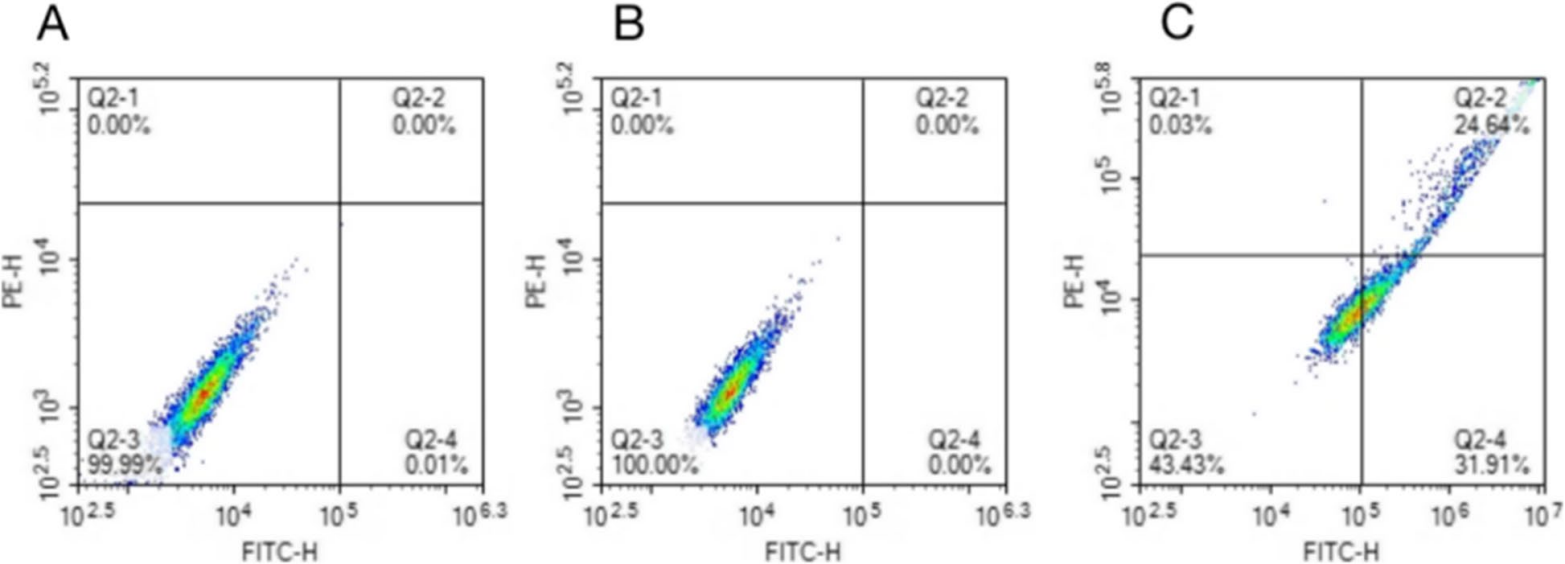
Flow cytology images of apoptosis in different group cells after transfection of siNUCKS1

**Fig. 6:**
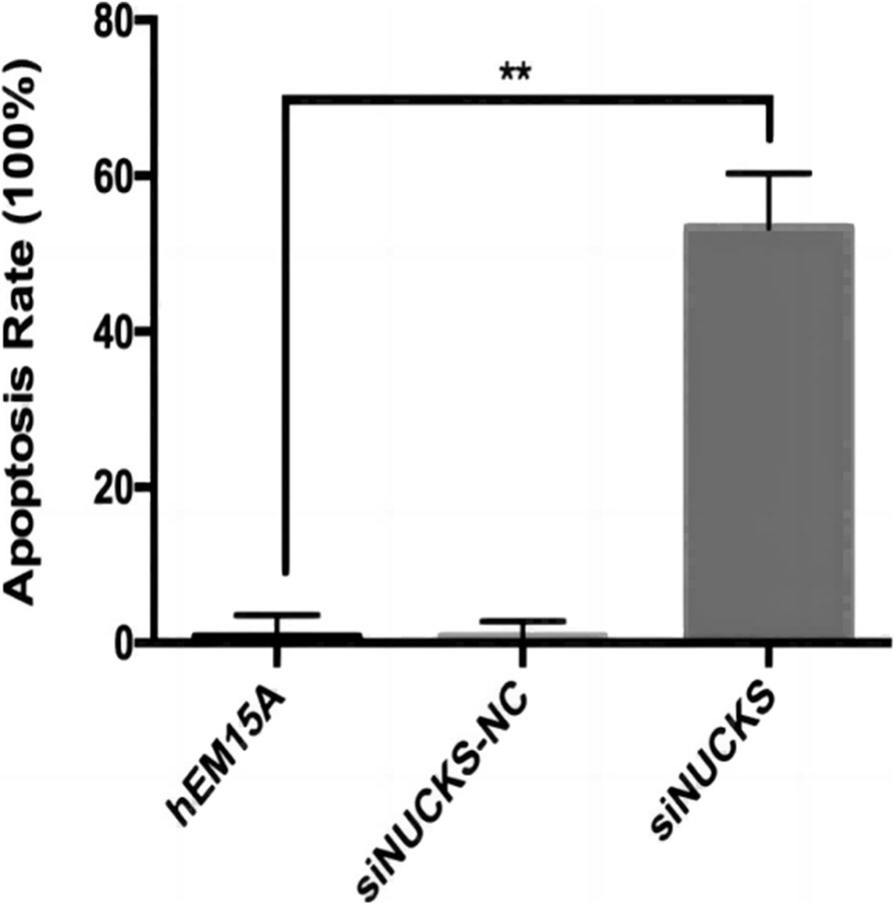
Apoptosis rate of siNUCKS1 transfected cells in each group.(*n*=3. Consistent with normal distribution. Differences between groups were compared using One-way analysis of variance. **P < 0.01)

**Fig. 7:**
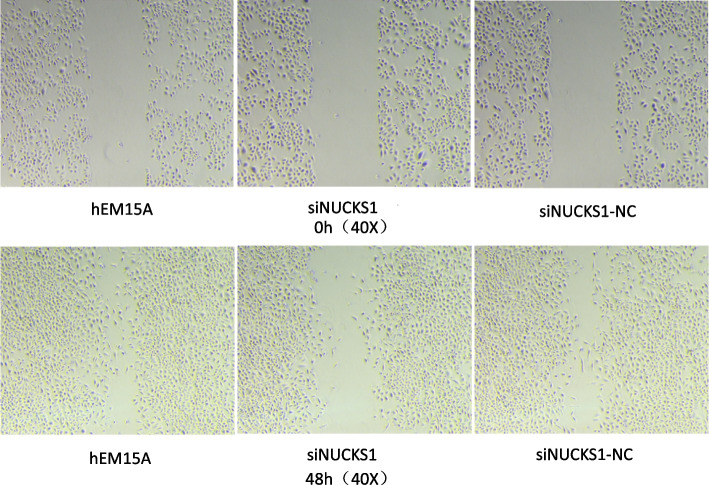
Cell migration images of each group after siNUCKS1 transfection

To ensure the inhibitory effect of siRNA, we selected three siRNA, and the expression of NUCKS1 in different groups was determined by RT-qPCR. SiNUCKS1-1 was most efficiently silenced than in blank controls and negative controls (Fig. 3). By CCK-8 assay, cell viability was significantly lower than in negative and blank controls (Fig. 4). Apoptosis was detected than negative and blank control groups through flow cytology (Figs. 5, 6). After 48 h of cell culture. The migration rate of hEM15A cells in the normal group was significantly faster than that of hEM15A cells in the NUCKS1-silenced group. The migration rate of hEM15A cells did not differ significantly from that of the normal group compared with the cell migration rate. It indicates that NUCKS has an inhibitory effect on the migratory ability of hEM15A cells (Figs. 7). In summary, cell function experiments demonstrated that the inhibition of siNUCKS1 decreased cell viability, increased apoptosis, and inhibited the capacity of migration.

### Expression of NF-κB, PI3K and VEGF in endometriosis

**Fig. 8:**
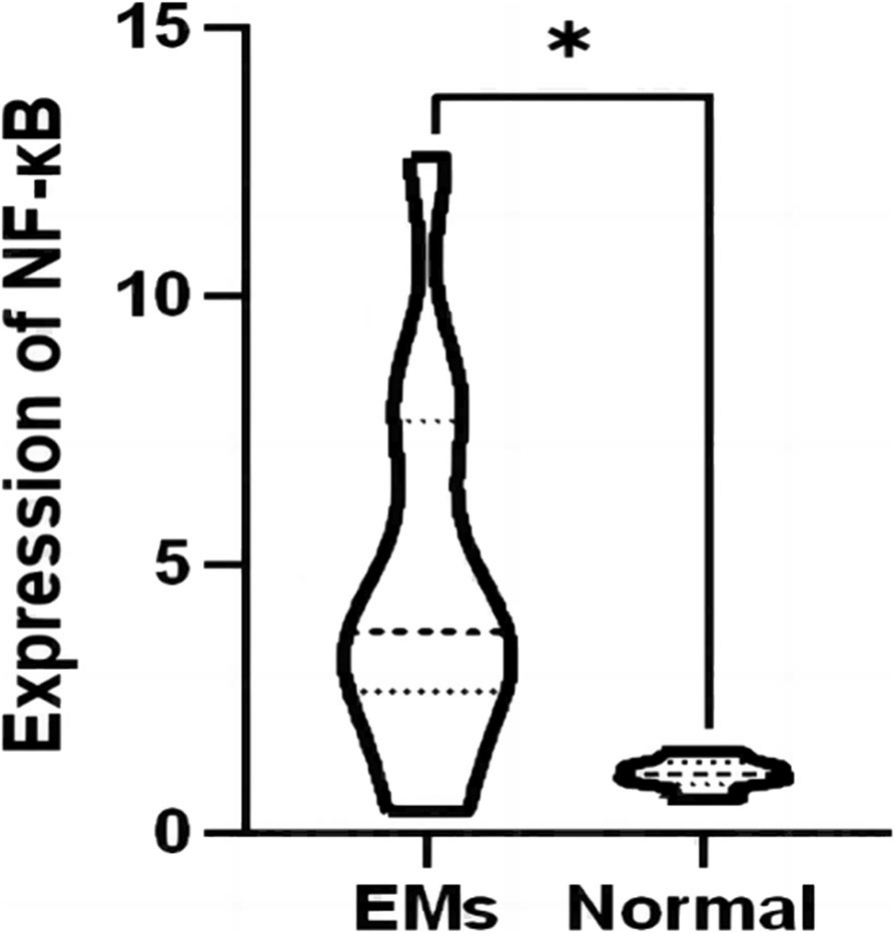
Expression of NF-κB mRNA levels in endometriosis tissues and normal tissues（EMs *n*=11，Normal *n*=6. Consistent with normal distribution. Differences between groups were compared using unpaired sample t test. *P < 0.1)

**Fig. 9:**
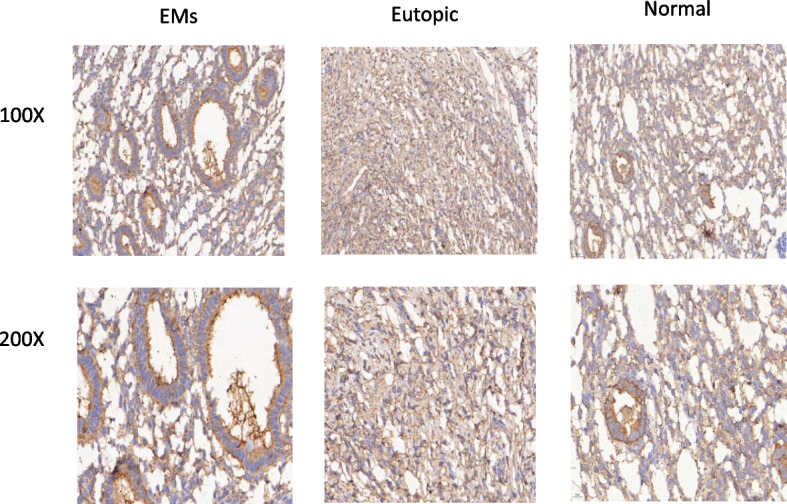
Immunohistochemical staining of NF-κB in endometriosis tissue, eutopic tissue and normal tissue

**Fig. 10:**
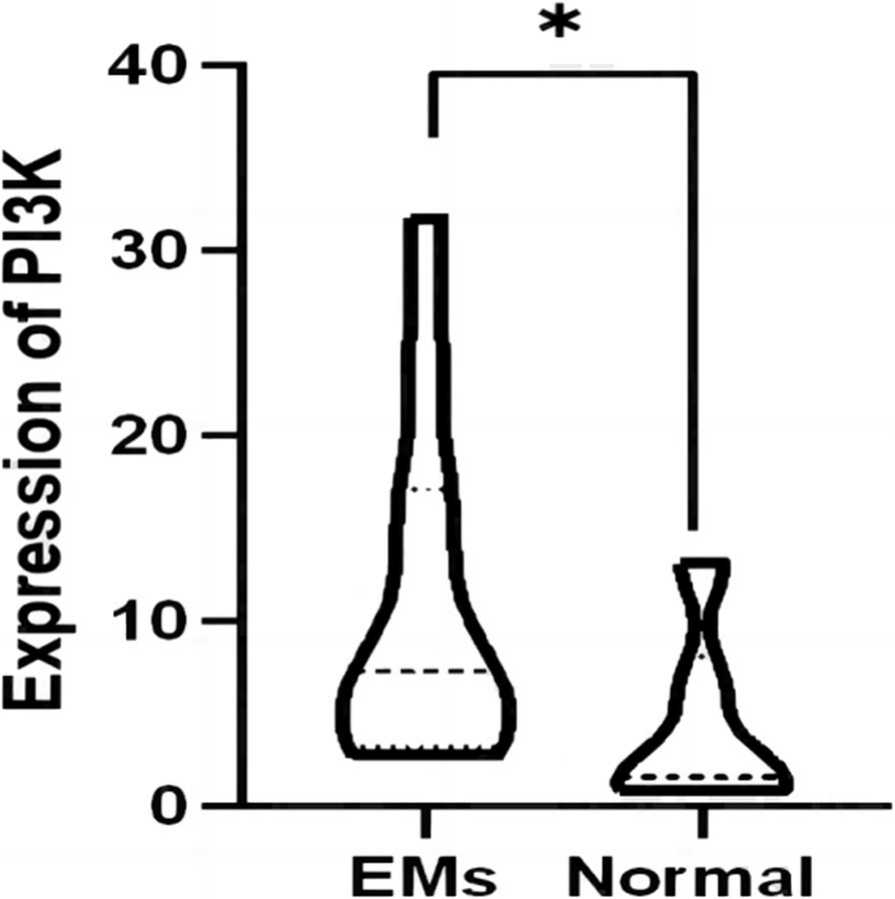
Expression of PI3K mRNA levels in endometriosis tissues and normal tissues.（EMs n=11，Normal *n*=6. Not consistent with normal distribution. Differences between the two groups were tested by Mann-whitney U test. *P < 0.1)

**Fig. 11:**
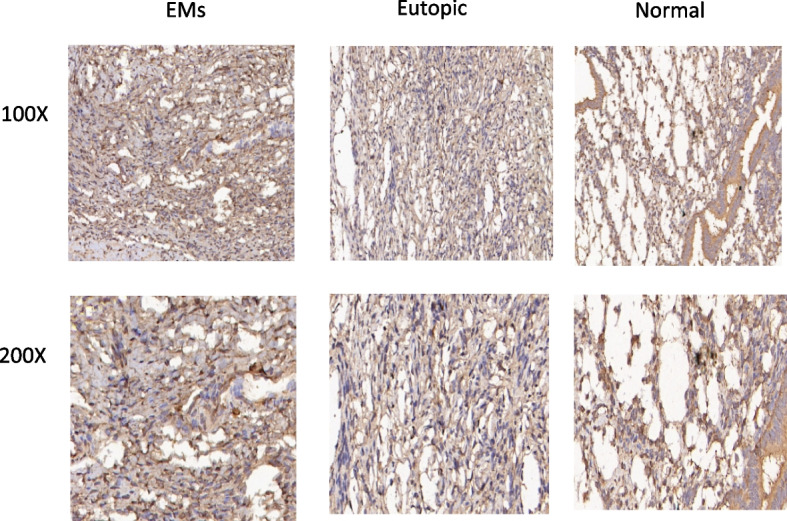
Immunohistochemical staining of PI3K in endometriosis tissue, eutopic tissue and normal tissue

**Fig. 12:**
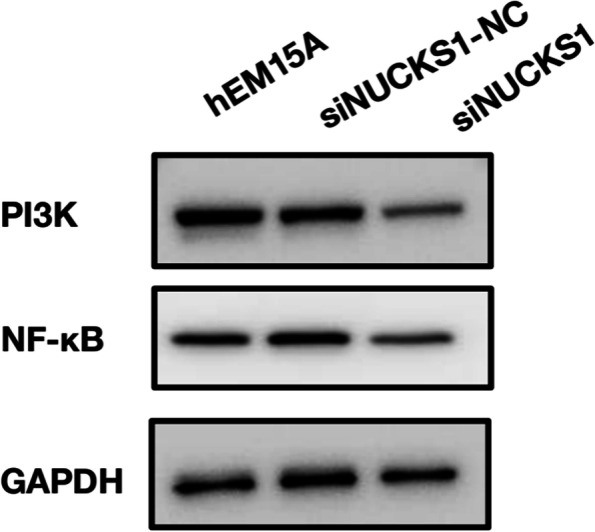
PI3K and NF-κB expression in each group cell after siNUCKS1 transfection. （the samples derive from the same experiment and that gels/blots were processed in parallel. See supplementary figures for details)

**Fig. 13:**
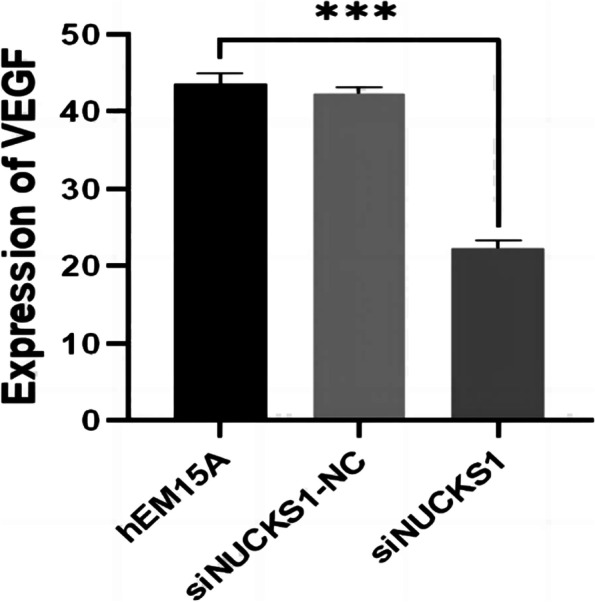
VEGF expression in each group cell after siNUCKS1 transfection. (n=3. Consistent with normal distribution. Differences between groups were compared using One-way analysis of variance. ***P < 0.001)

By RT-qPCR and IHC of the tissue samples, the expression of the NF-κB and PI3K in ectopic endometrial tissues was significantly increased compared to the control groups (Fig. 8, 9, 10, and 11). After inhibition of NUCKS1 in hEM15A cells, a significant decrease in NF-κB and PI3K expression was detected by western blot (Fig. 12). By ELISA, we found that the expression of VEGF in NUCKS1 inhibited cells was significantly decreased, and the difference between negative control and blank control group was not statistically significant (Fig. 13).

## Discussion

There are three main types of endometriosis: ovarian, peritoneal and deep nodal. This study mainly studied ovarian endometriosis. At present, most scholars believe that EMs is due to endometrial ectopic implantation, but Lang Jinghe, a famous scholar in China, proposed the eutopic endometrial determinism. He pointed out that the “3A” mode (Attachment, Aggression, Angiogenesis) of EMs. This model proposed that the refluxed endometrium needs to break through the inflammatory factors in the ascites, the immune cells in the peritoneal cavity and the extracellular matrix of the peritoneum to adhere to the extraendometrial tissue before further invasion and vascular. Subsequently, it was found that the expression levels of inflammatory factors such as transforming growth factor beta (TGF-β) IL-1, IL-6, IL-8, and IL-17 in peritoneal fluid, blood and endometriotic lesions differed between patients with and without EMs [11]. It was suggested that these inflammatory factors may have a coordinated role in the development of endometriosis [12]. In recent years, it has been suggested that EMs is a systemic auto-inflammatory immune disease and that some immune cells such as macrophages and lymphocytes secrete inflammatory mediators such as IL-8, tumor necrosis factor α (TNF-α), and VEGF that are involved in the pathogenesis of EMs [13]. Accumulating evidence suggests that immune cells, adhesion molecules, extracellular matrix metalloproteinase and pro-inflammatory cytokines activate/alter peritoneal microenvironment, creating the conditions for differentiation, adhesion, proliferation and survival of ectopic endometrial cells [14, 15]. Some inflammatory factors recruit and stimulate ectopic endometrial cells to proliferate and release a large number of cytokines including a large number of inflammatory mediators. Positive feedback is involved in the proliferation, adhesion, invasion and angiogenesis of the ectopic endothelium, promoting the progression of the disease. Among them, macrophages and their associated factors play a particularly important role in this process. Macrophages have been found to release a series of angiogenic factors, including VEGFa, IGF-1, IL-6 and bFGF. Angiogenesis is essential for the survival of ectopic endothelial cells and the formation of ectopic nodules. Endometriosis has similar biological characteristics to tumor, specifically, it has the characteristics of invasion, migration and susceptibility to recurrence. In recent years, NUCKS1 has been found to play an important role in the development and progression of many types of female reproductive cancers, such as ovarian cancer [16] and endometrial cancer cancer [17].

Nuclear Ubiquitous Casein and Cyclin-dependent Kinase Substrate 1 (NUCKS1) belongs to the HMG family, located on chromosome 1q32.1, and is widely present in almost all human cell types [5]. NUCKS1 acts as a substrate for second messenger-activated kinases, which mainly regulate several cellular activities such as replication, transcription, and chromatin condensation [6]. NUCKS1 was initially found to play an important role in glucose metabolism and maintenance of energy endostasis in vivo [18]. Later, an abnormal expression of NUCKS1 in many malignant tumors was found [17, 19, 20]. NUCKS1 is not only expressed at increased levels in malignant tumors, but also correlates with the cellular immune activity of tumors. Studies have found a positive correlation between IL-6, IL-12, lipocalin 2 ( LCN2) and NUCKS1 mRNA expression [21]. Subsequent studies found that NUCKS1 also plays an important function in some metabolic and inflammatory diseases. For example, NUCKS1 can control the cellular physiological functions through NF-κB and c-MYC regulatory signaling cascades [22, 23]. After knockdown of NUCKS1, some cells release some specific paracrine factors, mainly involved in inflammatory response, inflammation, regulation of cell proliferation, cell migration and secretion, and the expression of VEGFa was found to be elevated after knockdown of NUCKS1 [24]. And VEGF, IL-6, TNF-α and other inflammatory factors have very important roles in the development of EMs. However, NUCKS1 has not been studied in endometriosis.

Our research shows that NUCKS1 is highly expressed in endometriosis. This is consistent with studies of NUCKS1 in a variety of tumors as a cancer-like disease [7, 8]. To investigate the expression of NUCKS1 in endometriosis, which has similar characteristics with tumor, we collected ectopic endometrium and eutopic endometrium from patients with endometriosis, along with normal endometrial tissues. The expression of NUCKS1 in each group of tissues was detected by RT-qPCR. The results showed that NUCKS1 expression in ectopic, eutopic and normal endometrium tissues, but NUCKS1 expression in ectopic endometrial tissues was significantly higher than the eutopic and normal endometrium. This indicates that NUCKS1 is highly activated in the ectopic endometrial tissues of EMs patients, suggesting that NUCKS1 plays a very important role in the development and progression of endometriosis. Unfortunately, we failed to further investigate the relationship between NUCKS1 and clinicopathological parameters, such as stage, fertility index, cyst size, and degree of adhesion. We will further improve this part in the subsequent experiments. Subsequently, we performed functional tests through the Immortalized cells of eutopic endometrial mesenchymal cells from human endometriosis patients, including CCK8 assay, flow cytometry and transwell assay. The functional test results are shown that inhibition of NUCKS1 decreased cell viability and capability of invasion, and increased apoptosis in endometriosis cells. Similarly, NUCKS can promote proliferation, progression, and invasion in tumors [16, 17]. It shows that NUCKS1 has some relationship with the occurrence and development of EMs.

We have verified that NUCKS1 is involved in the development of endometriosis, but the specific mechanism is unclear. Studies on lung cancer [9], breast cancer [25] and gastric cancer [26] have shown that NUCKS1 functions through the PI3K signaling pathway. In addition, study [22] showed that following alkali-injury, inhibition of NUCKS1 is accompanied by a decrease of NF-κB. Therefore, we selected two signaling pathway proteins, PI3K and NF-κB, for validation of the downstream mechanism. We found increased PI3K and NF-κB expression in EMs tissues through western blot. Reduced expression of PI3K and NF-κB in NUCKS1-inhibited endometriosis cells. All the above indicated that NUCKS1 may function through PI3K or NF-κB signaling in endometriosis.

As previously mentioned, both endometriosis and NUCKS1 are strongly associated with VEGF. Study [22] showed that the expression of VEGFa was found to be elevated after knockdown of NUCKS1. Therefore, we examined the level of VEGF in inhibitory NUCKS1 endometriosis cells via ELISA. The results showed that the expression level of cellular VEGF was decreased after the inhibition of NUCKS1. Thus NUCKS1 may regulate invasion of ectopic endometrial cells as well as angiogenesis of local lesions through the VEGF.

Last but not least, the endometriosis is most common in women during their childbearing period, which can easily lead to infertility. One reason is that endometriosis can cause poor oocyte quality [27]. There are reports in the literature that poor oocytes quality can be improve with inositols supplementation [28–32]. Gender-specific approaches and attention to psychological cues are also important in treatment [33].

## Conclusion

In summary, NUCKS1 was differentially expressed in ectopic endometrium and normal endometrial tissues of patients with EMs. Cell viability, invasion and apoptosis are associated with NUCKS1 in endometriosis. Moreover, the expression of the pathway signaling proteins PI3K and NF-κB and the inflammatory cytokine VEGF is also associated with NUCKS1. It showed that NUCKS1 has some relationship with the occurrence and development of EMs. In addition, NUCKS1 may function through PI3K and NF-κB signaling pathways, and the inflammatory cytokine VEGF is involved. It may provide some reference for clinical diagnosis, treatment and prognosis judgment of patients with EMs. However, the mechanism of endometriosis development is very complex, and whether NUCKS1 can be used as an independent factor in the diagnosis, treatment and prognosis of EMs needs to be further investigated by expanding the sample size and extending the follow-up time as well as exploring the specific molecular signaling.

### Supplementary Information

**Additional file 1.**

## Data Availability

All data generated or analysed during this study are included in this published article.

## Abbreviations

NUCKS1: The Nuclear Ubiquitous Casein and cyclin-dependent Kinase Substrate 1
EMs: Endometriosis
RT-qPCR: Real-time fluorescence quantitative PCR
