# Supplementary material for: Roles of increased NUCKS1 expression in endometriosis
Source: BMC Womens Health. 2023 Aug 15;23:432. doi: 10.1186/s12905-023-02563-1 (PMC10426139; doi:10.1186/s12905-023-02563-1)
Supplement: Supplementary file 1 — Additional file 1. [file 12905_2023_2563_MOESM1_ESM.pdf]

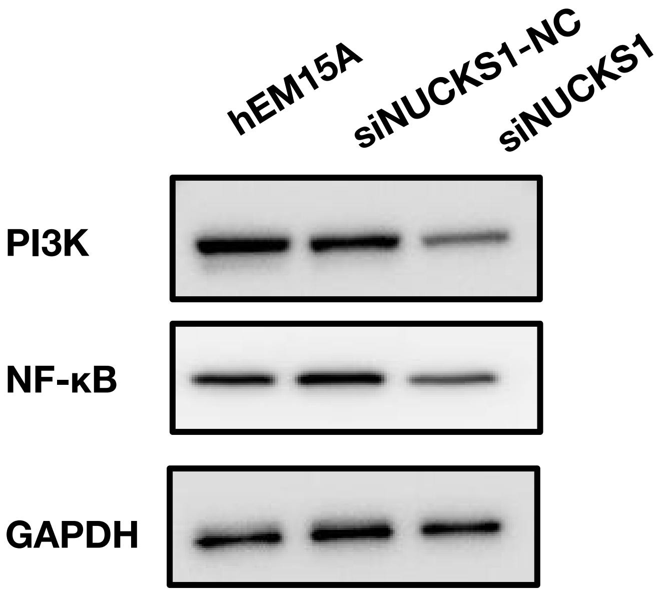

Figure 3-5 PI3K and NF-κB expression in each group cell after siNUCKS1 transfection

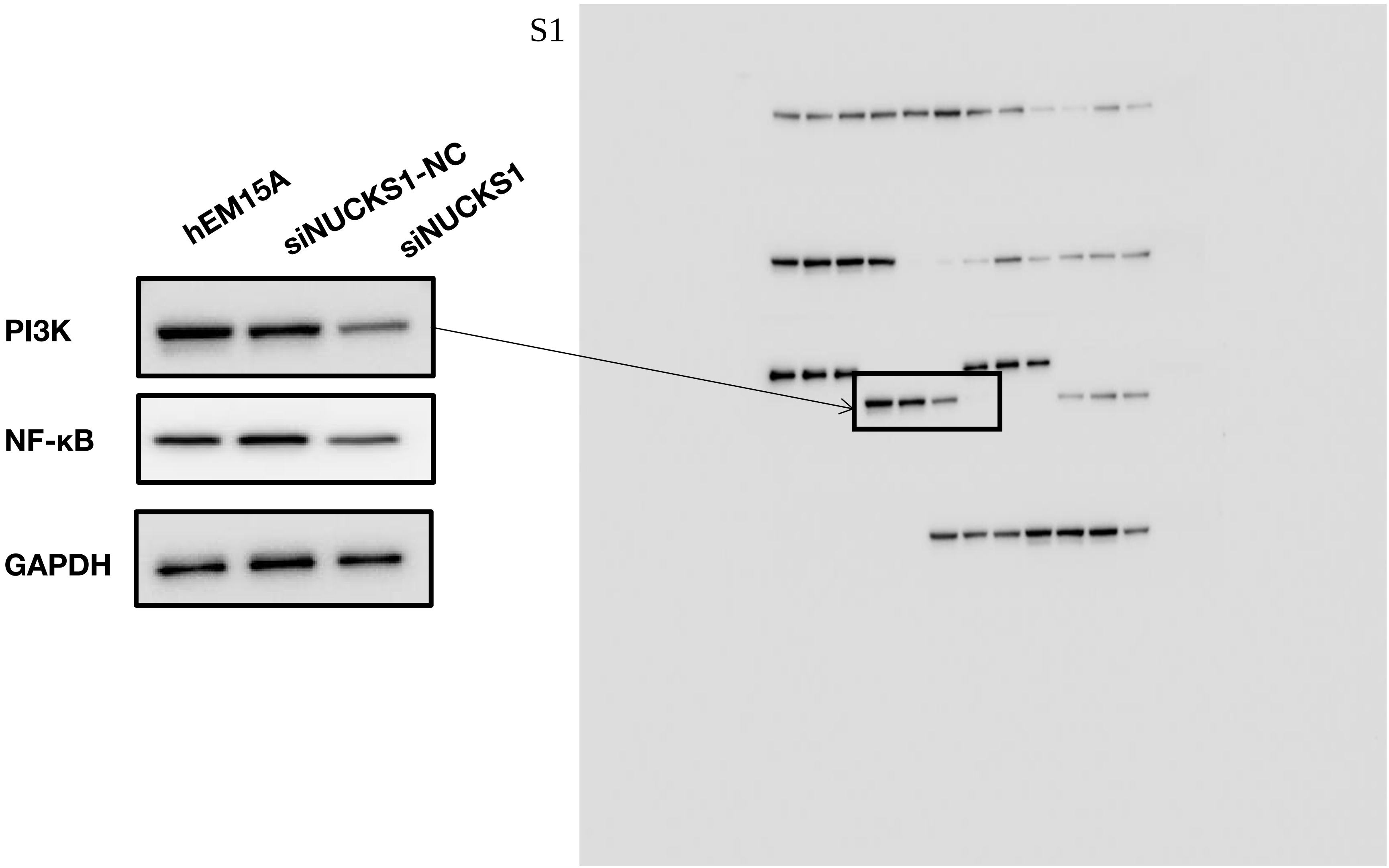

Figure S1 Repeat 1 for PI3K expression in each group cell after siNUCKS1 transfection

S2

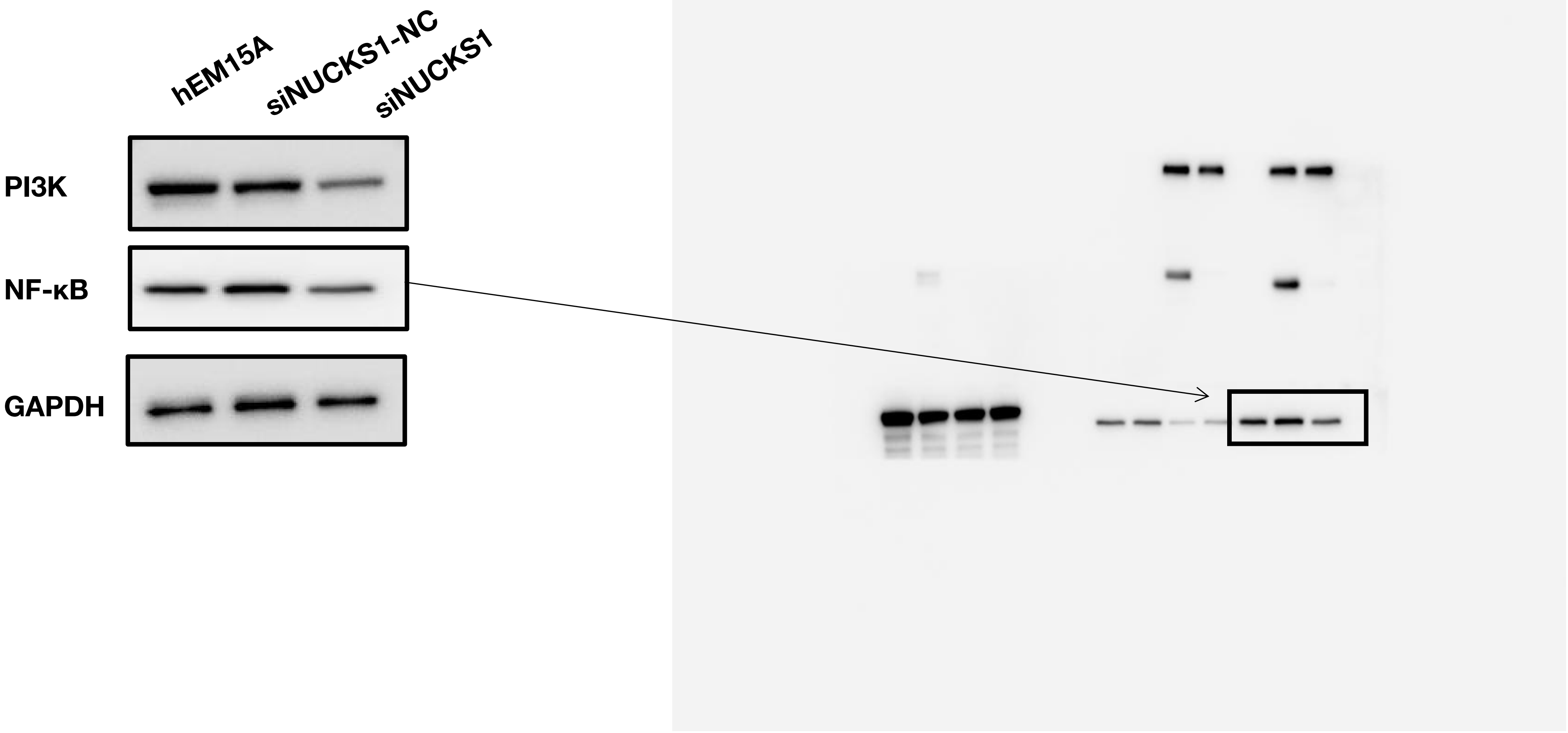

Figure S2 Repeat 1 for NF-κB expression in each group cell after siNUCKS1 transfection

S3

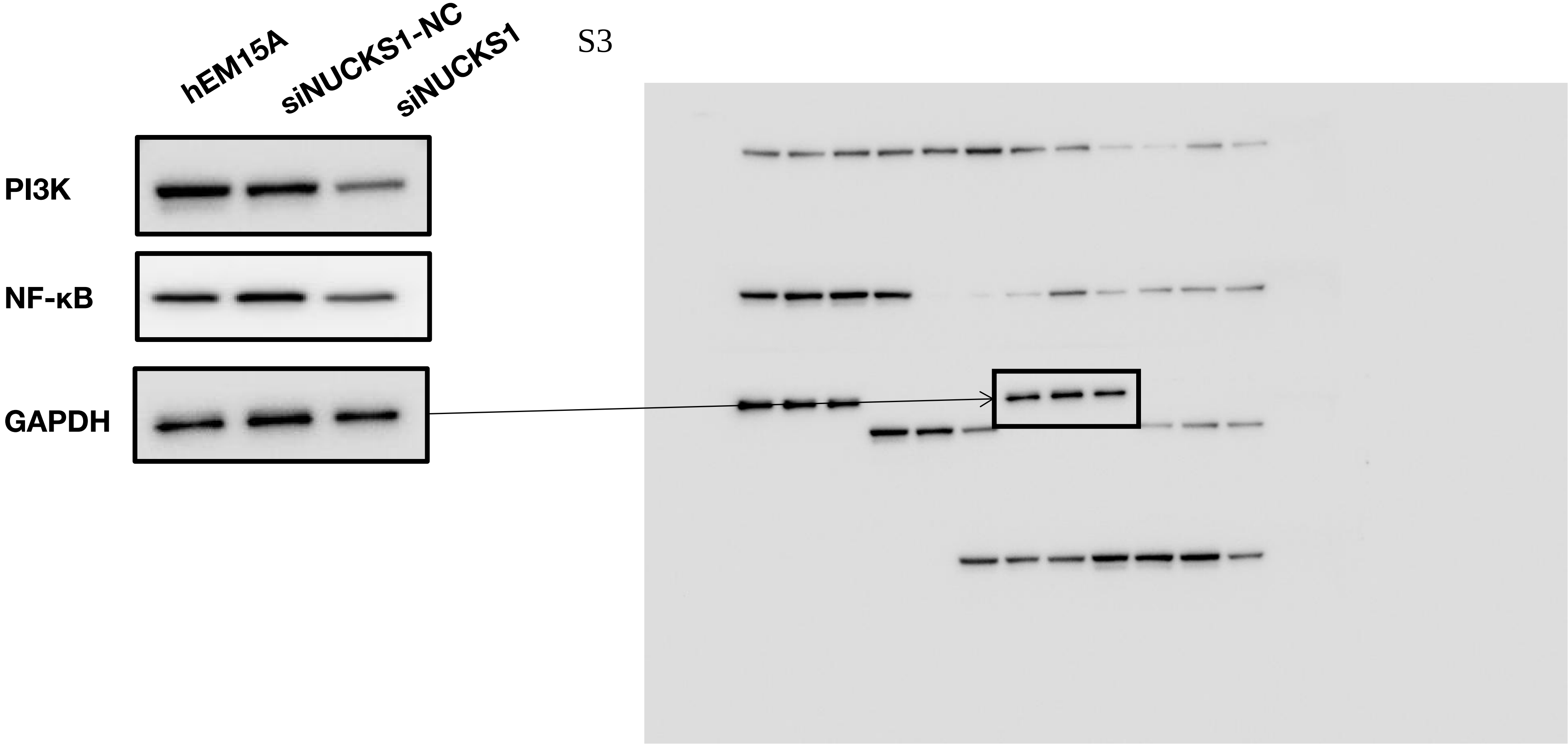

Figure S3 Repeat 1 for GAPDH expression in each group cell after siNUCKS1 transfection

Original picture：

S4

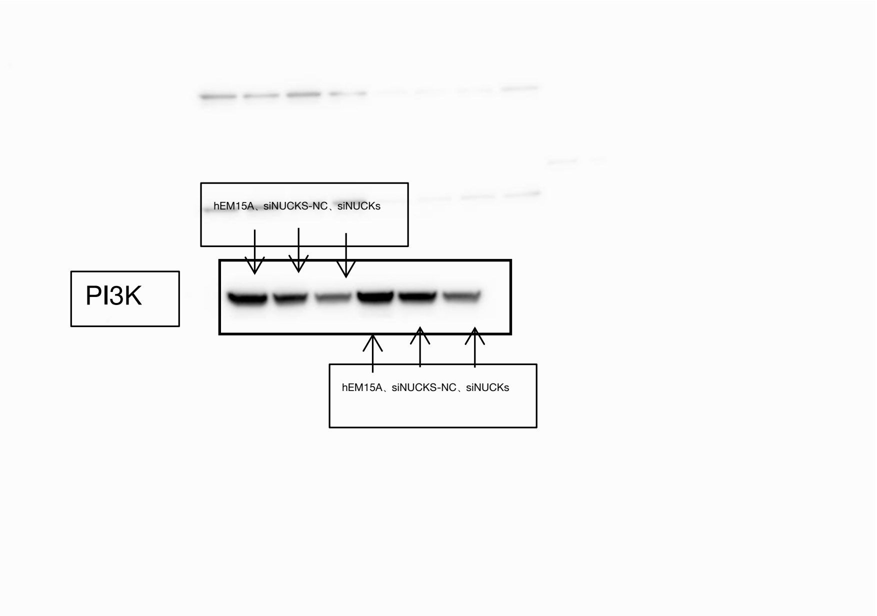

Figure S4 Repeat 2 and 3 for PI3K expression in each group cell after siNUCKS1 transfection

S5

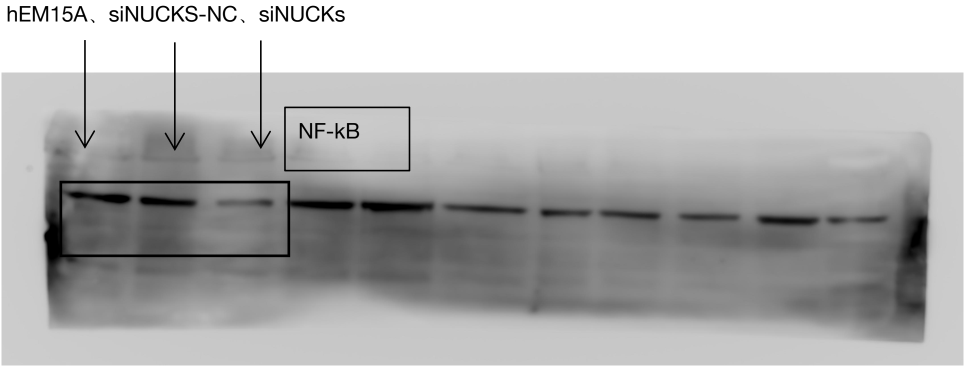

Figure S5 Repeat 2 for NF-κB expression in each group cell after siNUCKS1 transfection

S6

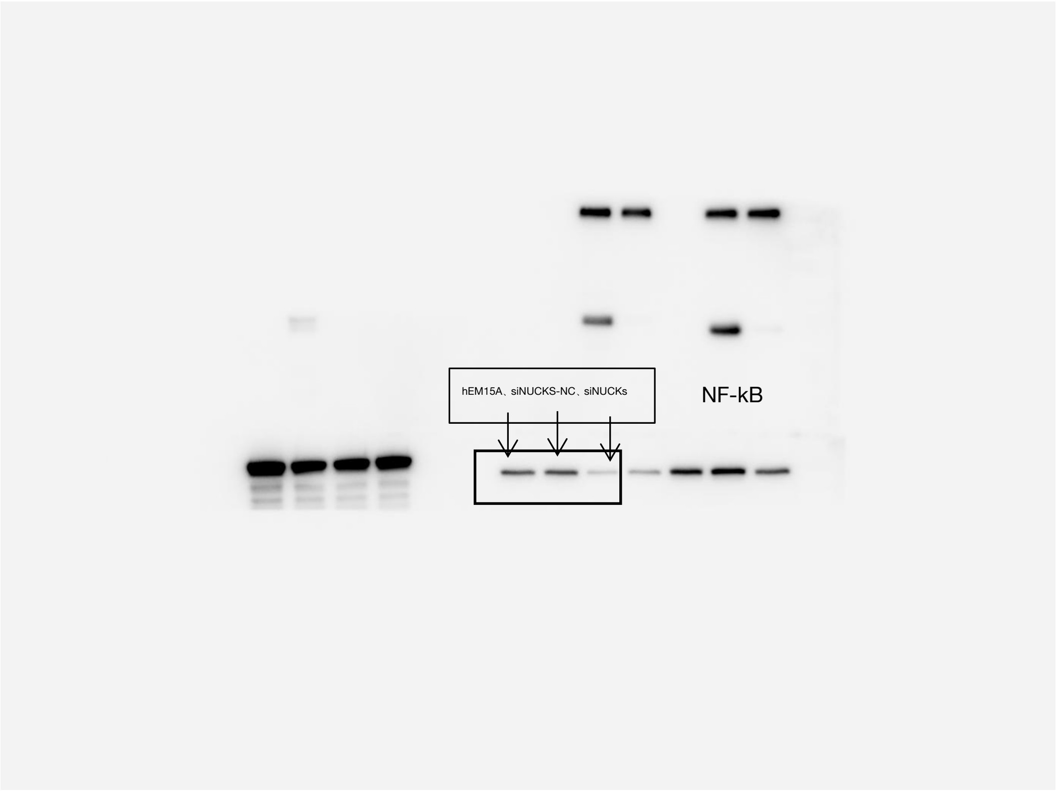

Figure S6 Repeat 3 for NF-κB expression in each group cell after siNUCKS1 transfection

S7

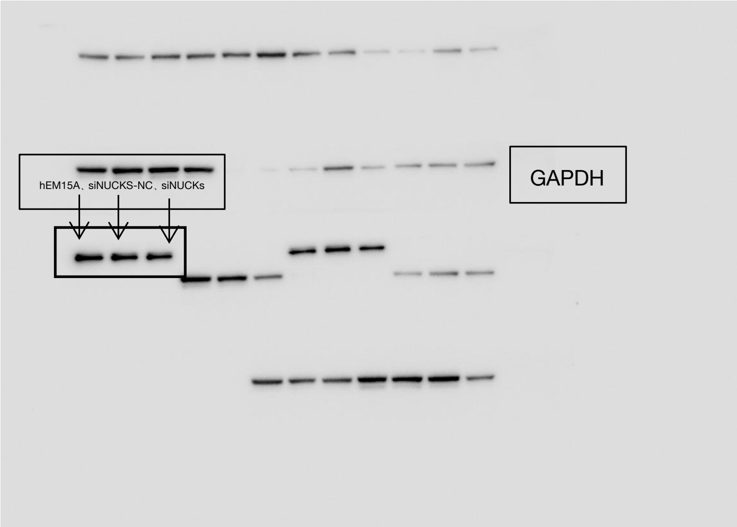

Figure S7 Repeat 2 for GAPDH expression in each group cell after siNUCKS1 transfection

S8

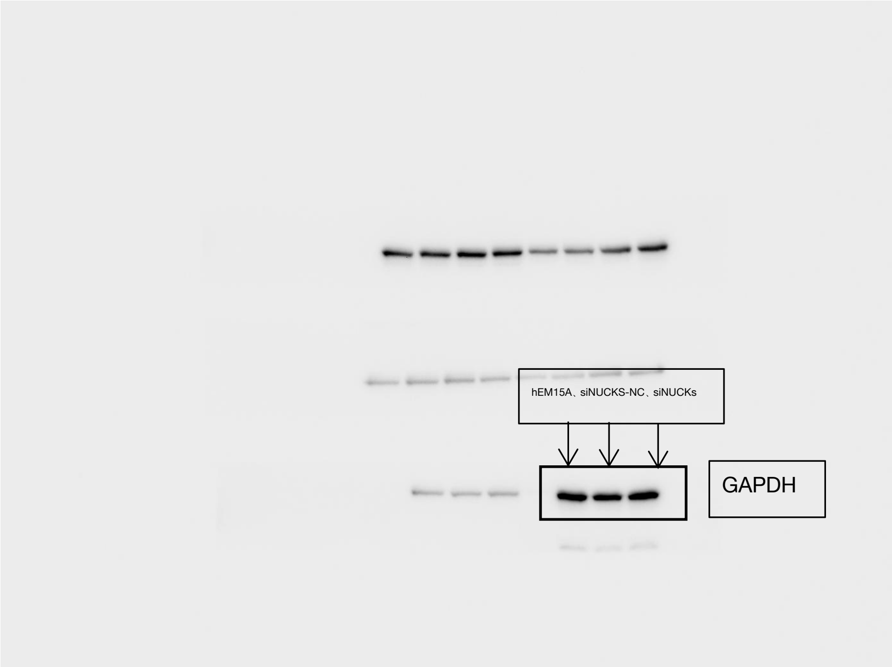

Figure S8 Repeat 3 for GAPDH expression in each group cell after siNUCKS1 transfection
